# Quantitative Analysis of Resorcinol from Marketed Hair Tonic Using Liquid Chromatographic Technique

**DOI:** 10.1155/2014/632591

**Published:** 2014-09-02

**Authors:** Amit Kumar De, Partha Pratim Chowdhury, Shyamaprasad Chattapadhyay

**Affiliations:** R&D Division, Deys Medical Stores (Mfg.) Ltd., 62 Bondel Road, Kolkata, West Bengal 700019, India

## Abstract

Quantitative estimation of resorcinol from marketed pharmaceutical formulation has been reported in this study. Resorcinol as a pharmaceutical ingredient has a broad spectrum of application but its application is limited due to its toxic side effects. Method for the accurate estimation of resorcinol is therefore essential. In the current study we have developed a chromatographic technique for its estimation from a marketed hair tonic meant for the treatment of several dermatological diseases of the scalp. A stainless steel column 25 cm in length and 4 mm internal diameter packed with octadecylsilane (5 *µ*m) was used for this purpose. The mobile phase was a mixture of phosphate buffer of pH 2.8 and acetonitrile. The flow rate was 0.6 mL·min^−1^ and the detection wavelength was 280 nm. The method was found to be linear between concentration range 10.28 *µ*g·mL^−1^ to 71.96 *µ*g·mL^−1^ with *r* ^2^ value 0.999. The accuracy of the method and the intraday and interday precession study presents the applicability of the method for the estimation of resorcinol from any pharmaceutical and cosmetic product containing resorcinol.

## 1. Introduction

Resorcinol (RC) or chemically Benzene-1,3-diol has got a wide range of applications in both chemical and pharmaceutical industry. As a medicine it has got a number of external applications and also as an antiseptic and disinfectant. Resorcinol is used in ointments for the treatment of chronic skin diseases like acne, seborrheic dermatitis, eczema, psoriasis, and other skin disorders. It is also used to treat corns, calluses, and warts. It is used in hair coloring agents as well. Resorcinol works by helping to remove hard, scaly, or roughened skin. It has got antifungal, antibacterial, and keratolytic properties. Despite having so many beneficial properties in large doses, it has got a number of side effects [1, 2]. Medical reports present long-term use of resorcinol in dermatological preparations that produce reversible adverse effects on human thyroid gland manifested as hypothyroidism [3, 4]. As such, it is essential to accurately quantify the amount of resorcinol in such formulations. The literature survey shows very few methods for the accurate estimation of resorcinol from these formulations [5–13]. Among these reported analyses, only a few presented the use of chromatographic technique for the estimation of resorcinol [14, 15].

Thus it is essential to develop an accurate and precise method for the estimation of resorcinol from cosmetic and pharmaceutical formulations. A hair tonic usually represents a formulation that contains a large number of components which are either a chemical compound or a compound obtained from natural source like* Pilocarpus pennatifolius*,* Emblica officinalis*, and so forth and many may have an oily base. The current study is based on the development of analytical procedure for estimation of resorcinol in presence of keratin hydrolysate, biotin, nicotinic acid, hyalkyl HBU (undecylenic acid derivative), povidone, ethanol, and glycerine. So development of an analytical method may be a bit challenging compared to other types of marketed formulation. The current literature survey fails to report a suitable analytical technique for the estimation of RC from hair tonics [16–18]. Also a spectrophotometric technique may fail to accurately estimate RC from such formulations that contain a large number of optically active components. The aim of this study is to develop a quick, accurate, and precise method for the estimation of resorcinol from marketed hair tonic.

## 2. Material and Methods

### 2.1. Materials

Waters C18 column (250 mm × 4.6 mm, 5 *μ*m) was purchased from Waters (Milford, MA, USA). The solvents used were of analytical reagent grade and for chromatography of HPLC grade and purchased from Spectrochem India Limited. Phosphoric acid of HPLC quality (Spectrochem, India) was used for pH adjustment of the eluent. RC (purity = 99.98%) was purchased from Sigma Aldrich India Ltd. The hair tonic containing RC as one of the principle components was purchased from market with batch number HV1113, manufacturing date January 2013, and expiry date June 2015.

### 2.2. Methods

#### 2.2.1. Instrumentation and Chromatographic Conditions

The chromatographic determinations were carried out on a Waters Alliance Separation Module (Waters, USA) quarternary gradient system and Waters 2489 dual lambda absorbance detector. The system control and data acquisition was carried out using Empower 3 software (Waters, USA). The separation was carried out in reverse phase Waters C18 column (250 mm × 4.0 mm, 5 *μ*; Waters, USA). The mobile phase was a mixture of 40% (v/v), 5.4 mM phosphate buffer (pH = 2.8) and 60% (v/v) acetonitrile which was filtered through 0.45 *μ*m Millipore filter paper. The flow rate was 0.6 mL/minute and the column was maintained at ambient temperature and the injection volume was 20 *μ*L. The detection wavelength was 280 nm. Prior to chromatographic separation both the standard and the sample were filtered through 0.2 *μ*m membrane filter (Pall Life Sciences, India).

#### 2.2.2. Preparation of Standard Solution and Sample Solution

**Figure 1:**
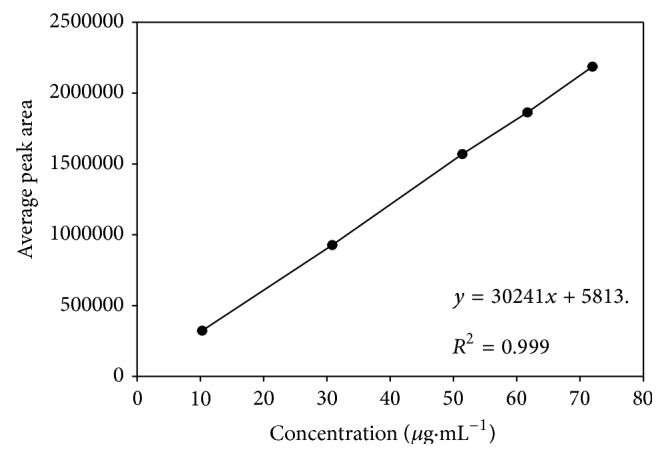
HPLC linearity curve for resorcinol.

*Standard Solution.* The RC stock solution (257 *μ*g·mL^−1^) was prepared by dissolving 12.85 mg of RC in 50 mL of 0.1 M hydrochloric acid solution by sonication. The stock solution was diluted to the range 10.28 *μ*g·mL^−1^ to 71.96 *μ*g·mL^−1^ of RC for analysis. A five-point calibration curve (Figure 1) was drawn for linearity study and for quantification purpose. Each dilution of the stock was injected in triplicates. The solution concentration and average peak area were presented in Table 1. The least square method was used for the curve fitting purpose. 

*Sample Solution.* 50 mL of sample containing 50 mg·100 mL^−1^ of RC was transferred accurately into a previously cleaned and dry 250 mL separating flask. 30 mL of 0.1 M hydrochloric acid solution was added and mixed. This solution was extracted with two 50 mL portions of chloroform. The layers on separation were collected separately and the chloroform layer was washed with another 15 mL of 0.1 M hydrochloric acid solution. Finally the aqueous layer and the washings were transferred to 100 mL clean and dry volumetric flask and the final volume was made up with 0.1 M hydrochloric acid solution. A portion of this solution was centrifuged at 10,000 rpm for 45 minutes. The supernatant was filtered and 5 mL of this solution was transferred in a clean and dry 25 mL volumetric flask and diluted to 25 mL with mobile phase.

#### 2.2.3. Validation of the Developed Method

The analytical method was validated as per USP [19] and ICH [20] guidelines using external calibration procedure. The studied parameters were accuracy, precision, linearity, range, ruggedness, and robustness. To ensure reliability and accuracy of the proposed method, recovery studies were carried out by mixing a known quantity of the standard drug with the sample at three different levels (80%, 110%, and 120% of assay value labeled as A, B, and C) with preanalyzed samples and contents were reanalyzed using the proposed method. Precession of the method was studied by making six injections of the standard solutions. The linearity of the method was established by triplicate injections of standard solution in the concentration range of 10.28 *μ*g·mL^−1^ to 71.96 *μ*g·mL^−1^. A five-point calibration curve (Figure 1) was drawn for linearity study and for quantification purpose. The intraday precision was calculated using six injections at the higher concentration range (50 *μ*g·mL^−1^) on the same day. These studies were repeated with the same solution on different days to obtain the interday precision. The specificity of the method was studied on the basis of peak purity. For peak purity study the analysis was carried out using a photodiode array (PDA) detector in place of UV-detector mentioned earlier. All other chromatographic conditions were kept unaltered. The limit of detection (LOD) and the limit of quantitation (LOQ) were determined from injections of progressively low concentrations of standard solution under optimized chromatographic conditions [21]. Ruggedness of the method was studied by carrying out experiment on instruments from different manufacturers. The robustness of the method was determined by making slight changes in chromatographic conditions like composition (±5%) and pH (±0.1%) of mobile phase.

The statistical analysis was carried out on Sigma plot software (version 8.02 SPSS Inc., USA) and MS Excel 2007. The data were processed and recorded as mean ± standard deviation of replicate measurements.

## 3. Result and Discussion

**Figure 2:**
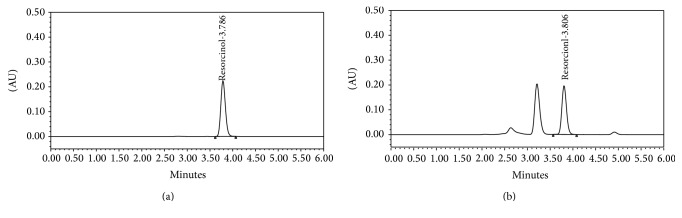
Representative chromatogram of standard (a) and sample (b) solutions under specified chromatographic conditions.

The average run time of analysis was only 6 minutes and the active component RC eluted at 3.8 minutes. There was sufficient resolution between the analyte peak and closely eluting peak as presented in the sample chromatogram (Figure 2(b)). The newly reported method is therefore less time consuming and more rapid compared to the few reported methods describing a chromatographic technique for analysis. The *λ*_max⁡_ for RC was found to be 280 nm; therefore, all analysis was carried out at this particular wavelength. The peak purity was determined by carrying out the analysis using a PDA detector. The results present the sample peak to be pure and spectrally homogeneous with peak purity angle 0.299 and the peak threshold 0.497. A reasonable resolution was observed between the sample peak and the closely eluting peaks as presented in Figure 2(b).

The average retention time for the RC peak was 3.78 ± 0.01 minutes (± SD; *n* = 3). The representative chromatograms in Figure 2 showed that the analyte peaks were symmetrical and well resolved from the closely eluting peaks.

### 3.1. Validation of the Developed Method

The present liquid chromatographic method was validated as per USP and ICH guidelines as discussed earlier. The results of the various parameters under study are presented as follows.

#### 3.1.1. System Suitability

System suitability was evaluated to verify if the chromatographic system was adequate for performing the analysis. The approximate results were theoretical plates (*N* = 6665.80), capacity factor (*k* = 1.54), peak asymmetry, or tailing factor (*t* = 1.12). The values for these parameters were satisfactory in accordance with the literature (Table 2) [20].

#### 3.1.2. Linearity and Range

The linearity of an analytical procedure is its ability to elicit test results that are directly, or by a well-define mathematical transformation, proportional to the concentration of the analyte in samples within a given range. The linearity was determined with standard solutions in concentration range 10.28 *μ*g·mL^−1^ to 1.96 *μ*g·mL^−1^. The curve fitting was linear with regression factor *r* ^2^ = 0.999 and equation *y* = 30241*x* + 5813 (Figure 1). The limit of detection (LOD) and limit of quantitation (LOQ) for standard solution was found to be 0.63 *μ*g·mL^−1^ and 1.92 *μ*g·mL^−1^ (Table 2). The LOD and LOQ of this method presented the sensitivity of the procedure within the range of analysis under consideration.

#### 3.1.3. Accuracy

The accuracy of analytical procedure is the closeness of test results obtained by that procedure to the true value. The accuracy of an analytical procedure is required to be established across its range. In our study the accuracy of the method was analysed on the basis of recovery study. In our analysis three different spiked solutions at concentration range 80%, 110%, and 120% of the calculated assay value of the sample was prepared. The 80% solution was prepared by appropriately diluting the stock solution and the 110% and 120% solutions were prepared by spiking the sample stock with known amount of the standard stock solution (solution A). The results presented a mean recovery of 100.33% with %RSD of 0.54 (Table 3).

The results present an acceptable accuracy over the range of study [20].

#### 3.1.4. Precession

The precession of an analytical procedure is the degree of agreement among individual test results when the procedure is applied repeatedly to multiple samplings of a homogeneous sample. The precession of an analytical procedure is usually expressed as the standard deviation or relative standard deviation of a series of measurements. In our study the precession was calculated on the basis of repeatability and intermediate precession. Six sample solutions at 100% of test concentration were separately prepared and analyzed using the analytical procedure under study. The intermediate precession was obtained by triplicate injections of the same solution on three different days. The intraday and interday precession values were calculated using the standard curve used for sample analysis (Figure 2). The intraday precession presented a %RSD value of 0.09 and that for interday varied from 0.01% to 0.08% only making the analytical procedure precise under the range of study.

#### 3.1.5. Specificity

As per ICH Q2/R1 guidelines specificity denotes the unequivocal assessment of the analyte under consideration from a mixture of components that may be expected to be present like impurities, degradation products, and matrix components. The specificity of RC from analysis of sample solution was studied on the basis of chromatographic peak purity study. This determines whether the peak was pure and free from any coelution of the matrix material with the analyte peak. A peak was considered spectrally homogeneous when the peak purity angle was less than threshold. In our study, the peak purity angle was 0.299 and the peak purity threshold was 0.497. The study therefore presents the peak to be spectrally homogeneous, free from any coeluting impurities, and specific for the analysis of RC.

#### 3.1.6. Robustness

The robustness of an analytical procedure was a measure of its capacity to remain unaffected by small but deliberate variations of procedural parameters listed in the procedure documentation and provide an indication of its stability during normal usage. In this study the robustness of the method was studied with variations of analyst, instruments, and solution stability on different storage conditions. The variations were also carried out in the quality of chloroform and acid used in the process. The results were found within tolerance limits (Table 4).

The results of this robustness study (Table 4) presented the method to be robust for the analysis of resorcinol from any formulation at a concentration as low as 1.92 *μ*g·mL^−1^ (Table 2).

## 4. Conclusion

The developed RP-HPLC method is simple, rapid, sensitive, precise, and selective for the estimation of RC from any formulation containing RC as one of its component. The method was validated in terms of linearity and precision in the studied concentration range as per ICH guidelines. The retention time of RC was only 3.8 minutes. Thus we can conclude that this method can be used for routine analysis, stability testing, and estimation of RC from various formulations available in the market.

## Figures and Tables

**Table 1:** HPLC linearity.

Concentration *μ*g·mL^−1^	Average Peak Area	Stadard Deviation
10.28	322582	3431.44
30.84	927071	584.54
51.40	1569200	613.22
61.68	1863087	7675.92
71.96	2186338	2417.10

**Table 2:** System suitability and validation data for the estimation of RC from hair tonic preparation.

Parameters	Resorcinol
System suitability	
Retention time (min)	3.78 ± 0.01
Capacity factor	1.54
Resolution	3.04
USP tailing factor	1.12
USP theoretical plates (*N*)	6665.80
Sensitivity	
Limit of detection (LOD) (*μ*g·mL^−1^)	0.63
Limit of quantitation (LOQ) (*μ*g·mL^−1^)	1.92
Precision	
Intra-day	
Repeatability (mg/100 mL) (*n* = 5) ± %RSD	45.80 ± 0.09
Inter-day	
Mean resorcinol content from hair tonic preparation (mg/100 mL) (day 1/day 2/day 3) (*n* = 3)	45.68/45.41/45.08
(%RSD) (day 1/day 2/day 3) (*n* = 3)	0.01/0.08/0.02

**Table 3:** Results of accuracy study.

% Of nominal value	Estimated Concentration of final solution in (*μ*g·mL^−1^)∗	Estimated Recovery %	Actual concentration of final solution (*μ*g·mL^−1^)∗	Average assay∗ (mg·100 mL^−1^)	Actual Recovery %	Accuracy %	%RSD
100	50.00	—	45.03	45.80	—	—	0.89
80	40.00	80.00	36.36	36.99	80.76	100.95	0.54
110	56.06	112.12	50.63	51.39	112.20	100.07	
120	60.66	121.32	54.69	55.55	121.29	99.97	

Note: ^*^Average from three replicate injections from sample preparations.

The recovery study is carried out over three-concentration range.

**Table 4:** Robustness of the developed procedure^**^.

Assay (mg/ 100 mL)											
Assay	Analysts			Instruments			Storage Condition			Chemicals used	
	1	2	3	I	II	III	20°C	30°C	45°C	Chloroform	Acid sol.
45.80	45.75	45.22	45.91	45.09	45.34	45.77	45.81	45.82	45.76	45.87	45.48

^**^Each analysis was carried out in three replicates.
